# Molecular characterization of a soybean *FT* homologue, *GmFT7*

**DOI:** 10.1038/s41598-021-83305-x

**Published:** 2021-02-11

**Authors:** Senhao Zhang, Mohan B. Singh, Prem L. Bhalla

**Affiliations:** Plant Molecular Biology and Biotechnology Laboratory, School of Agriculture and Food, Faculty of Veterinary and Agricultural Sciences, The University of Melbourne, Parkville, VIC 3010 Australia

**Keywords:** Plant sciences, Plant development, Plant molecular biology, Plant reproduction

## Abstract

Soybean (*Glycine max*) is a vital oilseed legume crop that provides protein and oil for humans and feedstock for animals. Flowering is a prerequisite for seed production. Floral transition, from vegetative to reproductive stage, in a plant, is regulated by environmental (light, temperature) and endogenous factors. In Arabidopsis, Flowering Locus T (FT) protein is shown to be a mobile signal that moves from leaf to shoot apical meristem to induce flowering. However, FTs role in soybean is not fully resolved due to the presence of multiple (ten) homologs in the genome. Two of the ten FT homologs (*GmFT2a* and *GmFT5a*) have a role in the floral transition while *GmFT1a* and *GmFT4* suppress soybean flowering. Recent deep sequencing data revealed that six *FT* homologs are expressed in shoot apical meristem and leaves during floral transition. One *FT* homolog, *GmFT7* showed strong expression during soybean floral transition. Though bioinformatic analyses revealed that *GmFT7* had high similarity with *GmFT2a,* ectopic *GmFT7* expression in *Arabidopsis* could not promote flowering or rescue the late-flowering phenotype of *Arabidopsis ft-10* mutant.

## Introduction

Soybean seed is one of the major sources of high-quality proteins and oil for human consumption and soybean seed meal for livestock feed. Further, soybean fixes atmospheric nitrogen through interaction with rhizobia reducing industrial fertilizer use and contributing to sustainable agriculture. Soybean is leading in oil production among the major oilseed crops with the world soybean production 361.0 million tons in 2018, accounting for 61% of the world oilseed^1^. Flowering is a prerequisite to yield of crops where seeds are the desired products. Moreover, with the climate change conditions, flowering is a critical factor for maintaining crop productivity.

Soybean flowering and seed maturity are determined by day length and temperatures during the growth and development stages. A wide range of soybean cultivars is grown worldwide across a range of latitudes with each cultivar adapted to a narrow latitude band restricting its cultivation. Maturity groups classification system (MGs) has been introduced to group soybean varieties based on their adaptation to specific environmental conditions. There are 13 maturity groups, namely, MG 000, 00, 0, and I to X. In North America, maturity groups MG 000, 00, and 0 are the earliest to flower and set seeds, and these maturity groups are mainly cultivated in south Canada^2^. In contrast, MGs IX and X are late flowering types and primarily grown in the southern US^3^.

Plants depend on their ability to measure the seasonal changes in photoperiod and ambient temperatures for their development and reproduction. Photoperiod is among important cues, which plants use to know the time of a year^4^. In 1920, Garner and Allard introduced photoperiod concept when they performed experiments with Maryland Mammoth cultivar of tabacoo^5^. Photoperiod is the extent of light and darkness in a 24 h cycle^6^. According to the responses under different photoperiods, flowering plants can be grouped as short-day (SD) plants, long-day (LD) plants, and day-neutral plants. The SD and LD plants flowering process can be accelerated if the day length is shorter or longer than a threshold. The flowering time of day-neutral plants is not regulated by day length. When photoperiod and temperature signals are perceived, shoot apical meristem (SAM) transition from vegetative growth to the reproductive stage to initiate flowering^7^.

Soybean is a short day (SD) plant implying floral induction will occur when plants are grown in a 12 h or less day length. Due to the critical role that flowering plays in determining seed yield, recent studies have focused on unravelling the molecular mechanisms underlying floral transition in soybean^8–18^. Most of our knowledge on flowering is based on a model plant, *Arabidopsis*. However, *Arabidopsis* is an LD plant requiring the longer than a critical day length for flowering.

In *Arabidopsis*, three main environmental pathways, namely photoperiod, vernalization, and autonomous control of flowering^19^. Of these, the photoperiod pathway being the most ancient and conserved. CONSTANS (CO), a transcriptional regulator, integrates day length information in the flowering photoperiod pathway. Furthermore, *CONSTANS* (*CO*) transcription itself is regulated by the circadian clock^20^. In brief, CO protein is stabilized by light and can only accumulate under inductive long days while degrading quickly in darkness^21^. *CO* is expressed in leaves, and it activates the expression of *FLOWERING LOCUS T* (*FT*). *FT* encodes a protein which is a mobile flowering signal^19^. FT protein, rather than *FT* mRNA, is the florigen which moves from leaf to shoot apex^22–24^. At SAM, FT interacts with FD (FLOWERING LOCUS D), a bZIP transcription factor to form FT-FD complex leading to initiation of flowering by activating floral integrator genes such as *SUPPRESSOR OF OVEREXPRESSION OF CO 1* (*SOC1*) and *LEAFY* (*LFY*) and enhances the expression of floral identity gene *APETALA1* (*AP1*) and represses the expression of repressor *TERMINAL FLOWER* (*TFL*). *AP1* is a homeotic gene that encodes a transcription factor to specify the floral meristem identity and determines sepal and petal development^25^. On the contrary, rice (*Oryza sativa*) is an SD plant and Heading date 3a (Hd3a), the *Arabidopsis* FT homologue in rice, interacts with 14-3-3 proteins first in SAM. The Hd3a-14-3-3 complex translocates to the nucleus to bind to OsFD1, an *Arabidopsis* FD homologue in rice, to activate floral transition^26,27^ highlighting differences between Arabidopsis (a model plant) and a crop plant.

Ten *FT* homologues found in the soybean genome and genetic studies showed that *GmFT2a* and *GmFT5a* promote flowering^12^ while *GmFT1a* and *GmFT4* function repressors^13,18^. Functions of *GmFT2a* and *GmFT5a* are non-redundant, and *GmFT2a* is more critical under SD condition, while *GmFT5a* is more critical under LD condition^9^. Mutagenesis of *GmFT2a* resulted in delayed flowering under both LD and SD conditions^8^, *ft2aft5a* double mutants exhibited late flowering even under SD condition^9^. Besides, *GmFT2a* and *GmFT5a* are also involved in post-flowering stem termination with GmFT5a being more effective than GmFT2a by interacting with a bZIP transcription factor GmFDL06^16^. In contrast, *GmFT4* was primarily expressed in leaves and showed induced and reduced expression under LD and SD conditions, respectively^18^. Highly elevated expression of *GmFT4* was observed in transgenic soybean plants over-expressing soybean maturity gene *E1,* which is a legume specific gene with a highly significant effect on flowering time and photoperiod sensitivity. In addition, ectopic expression of *GmFT4* resulted in late flowering phenotype in transgenic *Arabidopsis* plants. Like *GmFT4*, *GmFT1a* was primarily expressed in leaves, *GmFT1a* showed induced and reduced expression under LD and SD conditions, respectively^13^. Over-expression of *GmFT1a* in soybean delayed flowering, confirming that it as a flowering repressor^13^. Furthermore, Chen et al.^10^ examined *GmFT2b* sequence variations in 195 soybean cultivars and found that a Hap3, a major halophyte correlated with significant earlier flowering in higher altitude soybean cultivars.

Previous deep sequencing^28^ studies in our lab to characterize leaf and shoot apical meristem transcriptome in soybean after short day treatment to evoke floral transition revealed a soybean *FT* homologue, *Glyma02g07650*, named to *GmFT7* here and after. Differential gene expression analysis identified the genes involved during soybean floral transition. *GmFT7* was found to be highly expressed both in leaf and shoot apical meristem tissues upon short-day treatment. This study aimed to understand the function of *GmFT7* during floral transition.

## Results

### Identification, phylogenetic and expression analysis of *GmFT7*

FT is a member of the Phosphatidylethanolamine-binding protein (PEBP) gene family encoding a 175 amino acids (aa) in Arabidopsis (AtFT). Most of the soybean homologues (GmFT homologues) are predicted to be between 172 to 176 aa. A database search showed that *GmFT7* (*Glyma02g07650*) has a CDS region of 195 bp, corresponding to 64 amino acids. The predicted GmFT7 protein with a molecular weight of 7.50 kDa is much smaller than that of GmFT2a (19.74 kDa), GmFT5a (19.09 kDa) and *Arabidopsis* FT (19.81 kDa), but with a higher isoelectric point (theoretical pI) of 10.38 than GmFT2a, GmFT5a and AtFT (5.59, 7.85 and 7.75 respectively). The instability index ranging from 41.27 (GmFT2a) to 48.81 (AtFT) indicated that all the four FT proteins are unstable. GRAVY (Grand Average of Hydropathy) values, which indicate hydropathicity of proteins, are all negative implying that all the four FT proteins are hydrophilic. Protein subcellular location analysis showed that GmFT7 is located in cytoplasm and nucleus similar to GmFT2a/5a and *Arabidopsis* FT.

**Figure 1:**
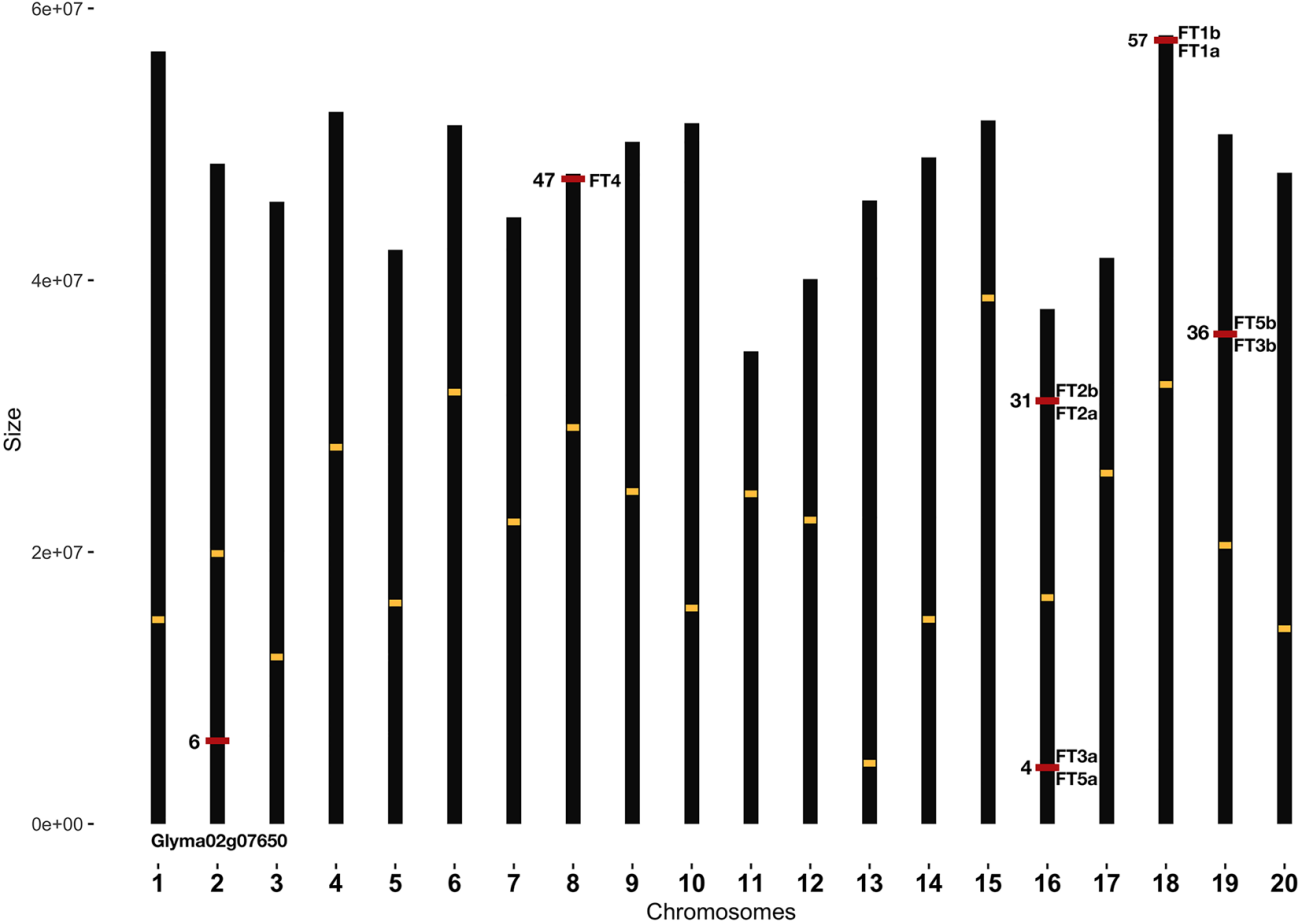
Chromosomal locations of the soybean *FT (GmFT* homologues) genes. The *FT* genes were mapped to 5 out of 20 chromosomes. Bars represent 20 chromosomes in soybean and are scaled to chromosome sizes. Size is in a million bases. The number on the left side of the red line indicates the position on a chromosome. Yellow boxes on each chromosome represent the centromere position.

**Figure 2:**
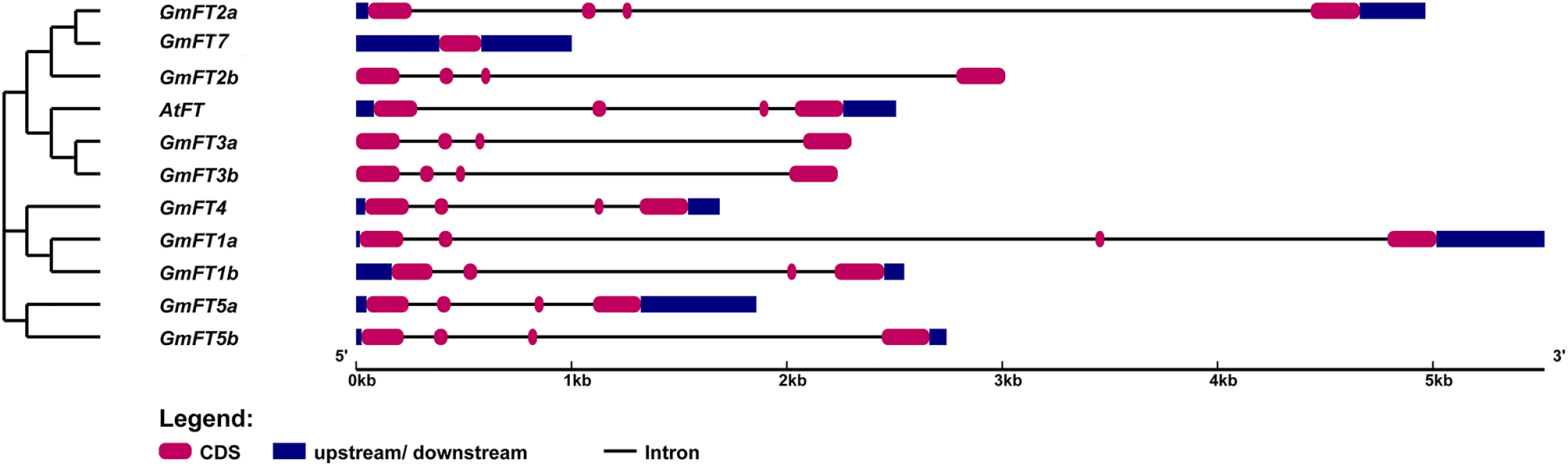
Phylogenetic relationship and gene structures of soybean *FT* genes, *GmFT* homologues. Gene structures of ten soybean *FT* homologues and *Arabidopsis FT* were illustrated using GSDS2.0 server (http://gsds.cbi.pku.edu.cn/). The red box represented CDS; blue rectangles represented up- or down-stream elements; introns were shown as solid lines. For a phylogenetic tree, sequence alignment was performed using MUSCLE program in MEGA7 with default parameters. Maximum Likelihood tree with cpREV + G substitution model was constructed with the Bootstrap method of 1000 replicates.

Chromosomal distribution of ten soybean *FT* homologues showed that all *FTs* are localized on five of the 20 soybean chromosomes, *GmFT1a/1b* on chromosome 18, *GmFT2a/2b/3a/5a* on chromosome 16, *GmFT3b/5b* on chromosome 19, *GmFT4* on chromosome 8 and *GmFT7* on chromosome 2 (Fig. 1). Centromeric coordinates for each chromosome were retrieved from Golicz et al.^29^. Among soybean *FT* homologues, eight *FT* homologues found in pairs (*GmFT1a*/*GmFT1b*; *GmFT2a*/*GmFT2b*; *GmFT3a*/*GmFT5a*; and *GmFT3b*/*GmFT5b*) except *GmFT4* and *GmFT7* which are located on chromosomes 8 and 2, respectively (Fig. 1). Gene structures of *GmFTs* showed that all the *FTs* have four exons except that *GmFT7* has only one (fourth) exon (Fig. 2). Previous studies showed a significant difference between *FT2c* from domesticated soybean (*Glycine max*) and wild soybean (*Glycine soja*), where *GsFT2c* is an intact *FT* homologue while *GmFT2c* harboured a structural rearrangement in domesticated soybean^17,30^. This variation suggested that there is no intact *FT2c* in domesticated soybean. A 13.6 kb insertion separated *GmFT2c* into two portions with the C-terminus containing only the fourth exon and 3′-UTR^17^. Our analysis showed that *GmFT7* has only one exon in the C-terminus, that shows 100% of sequence similarity to the fourth exon of *GsFT2c* (Supplementary Fig. S1).

To characterize phylogenetic relationships between GmFT7 and other FT homologues in soybean, protein sequences of GmFT7 and other GmFT homologues were retrieved from Phytozome. We also included *Arabidopsis* FT in the analysis. Ten GmFT homologues were classified into four groups and one singleton (GmFT4; Supplementary Fig. S2) under the Maximum Likelihood tree. FT homologues in the same clade showed a higher percentage of identity: 98.41% between GmFT2a and GmFT7, 96.51% between GmFT2a and GmFT5a, 94.29% between GmFT3a and GmFT3b, 90.91% between GmFT2a and GmFT2b, while a relatively lower identity between GmFT1a and GmFT1b.

**Figure 3:**
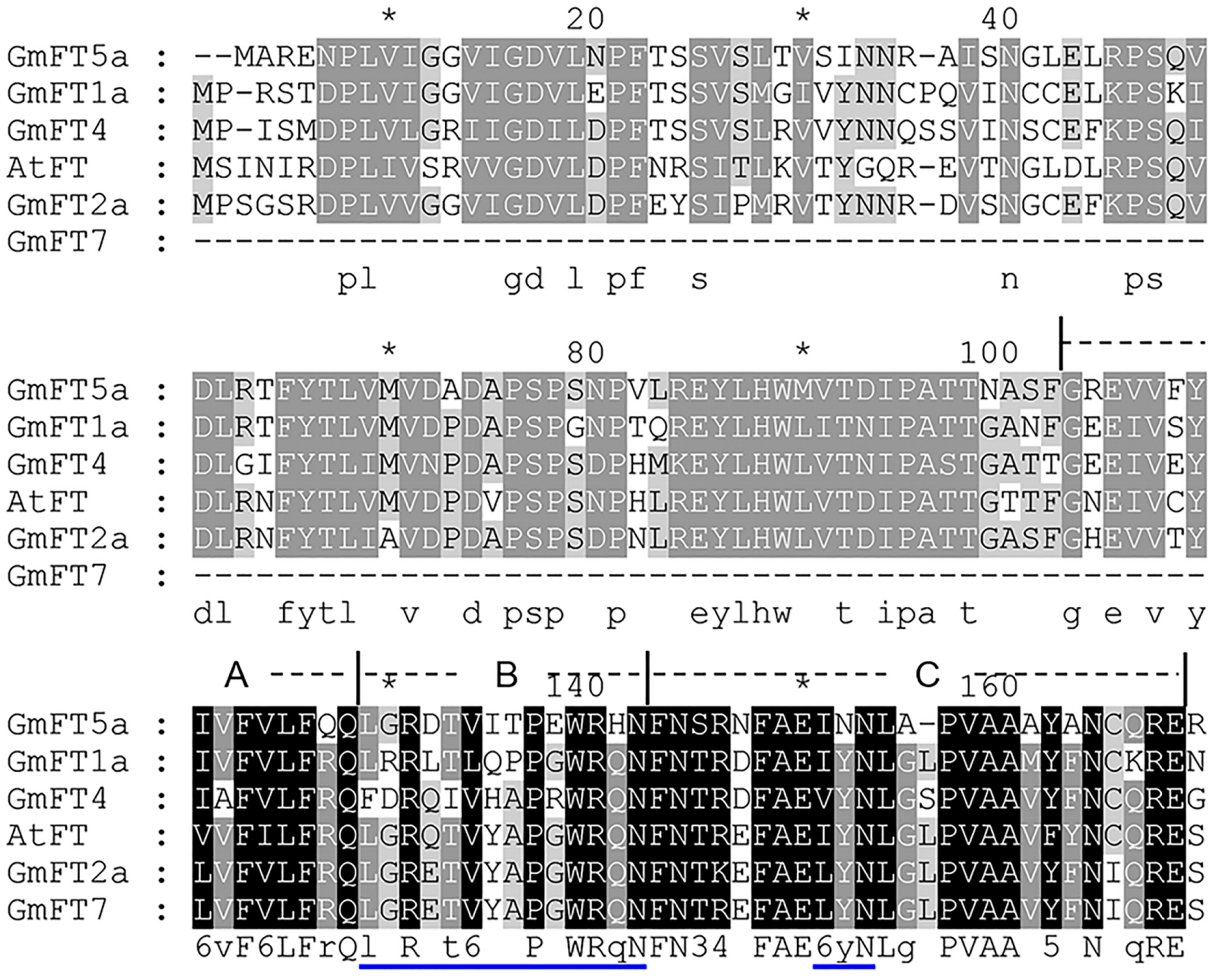
Multiple sequence alignment of *Arabidopsis* FT and soybean FT homologues (GmFT1a, GmFT4, GmFT2a, GmFT5a, and GmFT7). The alignment was performed using Clustal Omega (https://www.ebi.ac.uk/Tools/msa/clustalo/). GeneDoc was used for shading. Segments A, B, and C are indicated. Blue lines indicate critical amino acids in FT.

Gene structure analysis showed that *GmFT7* only has the fourth exon compared to other *FT* homologues with four exons (Fig. 2). The fourth exon had been shown to harness a critical role^31^. Protein sequence alignment of GmFT7 and the four well studied GmFT homologues (flowering promoters and repressors) showed that GmFT7 has a conserved 14-amino acid segment B and the LYN in segment C^12^ (Fig. 3). The second last amino acid in segment B was indicated as a critical amino acid that was regarded as FT proteins^31^, suggesting that *GmFT7* can function as an *FT*. LYN (triad) is a three amino-acid region which distinguishes FT and TFL1 proteins. This triad is more conserved in FT compared to TFL1. The first residue of the triad is leucine (L) or isoleucine (I), followed by tyrosine (Y) and asparagine (N)^31^. In Arabidopsis FT and soybean GmFT5a this triad is IYN. However, it is LYN for GmFT2a and GmFT7.

**Figure 4:**
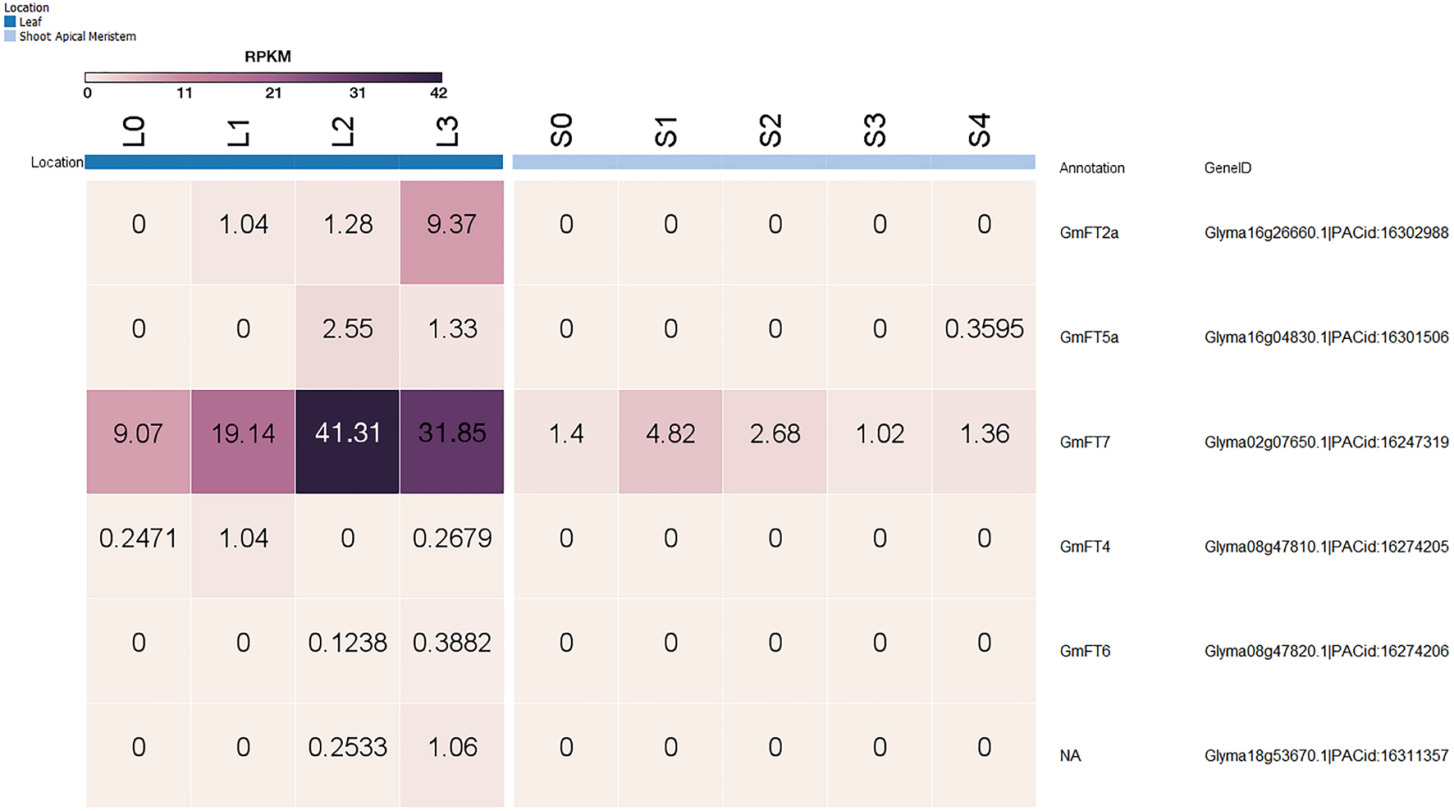
The expression profiles of soybean *FT* mRNAs. The heatmap was produced using RNA-seq data generated in our previous study^23^. Values are in RPKM (Reads Per Kilobase of a transcript, per Million, mapped reads). Samples: leaf from 0 short-day (L0) to 3 short-day (L3) and shoot apical meristem from 0 short-day (S0) to 4 short-day (S4). Heatmap was constructed using GENE-E (https://software.broadinstitute.org/GENE-E/).

**Figure 5:**
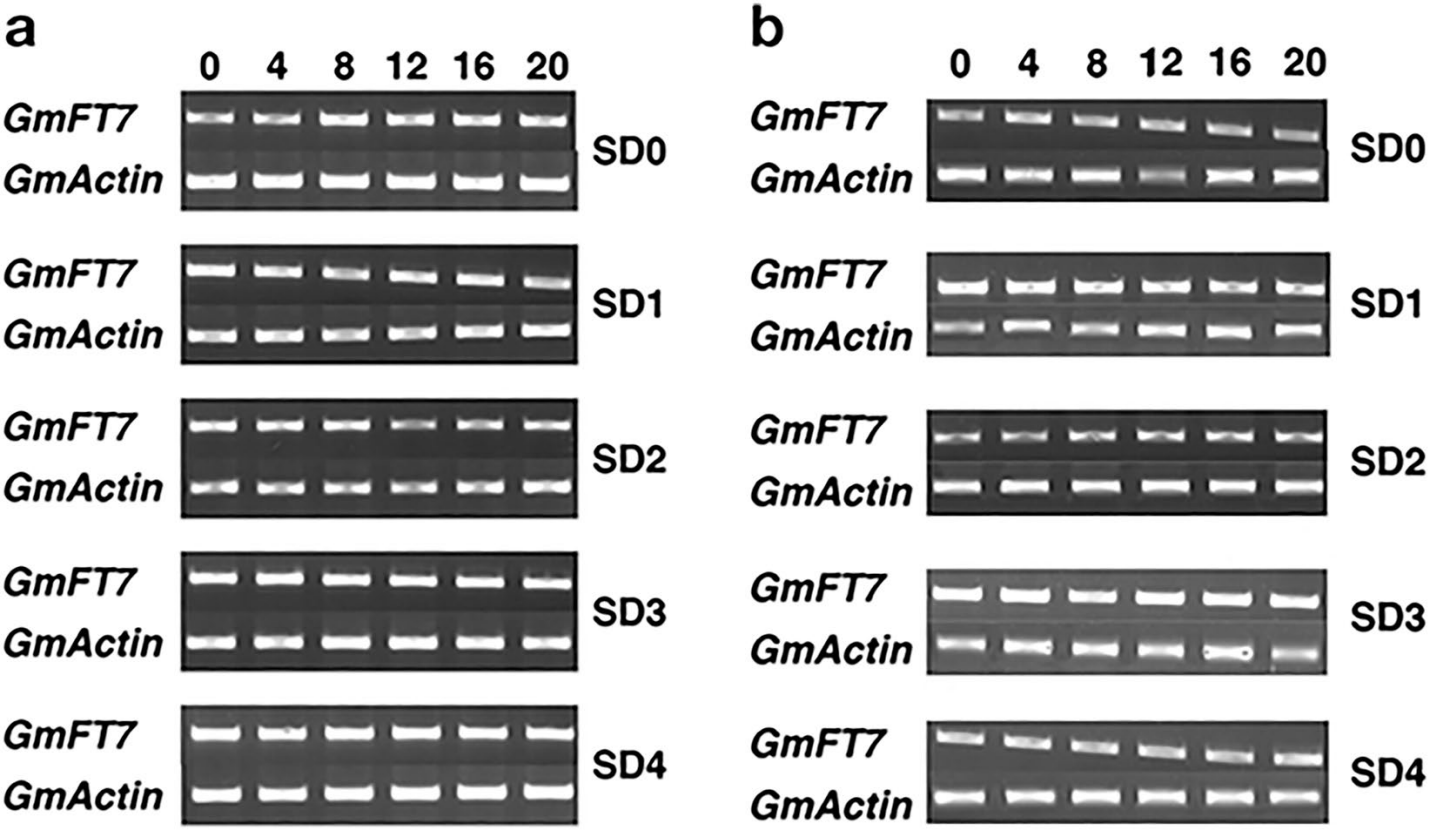
*GmFT7* mRNA expression in soybean leaf (**a**) and shoot apex (**b**) under SD condition (8 h light/16 h dark). *GmActin* was used as a reference gene. 0, 4, 8, 12, 16, 20 indicates zeitgeber time (ZT); samples were collected every 4 h. Each time point represents pooled samples from 5 plants. SD 0–4, days under SD condition.

Our earlier RNA-seq experiment highlighted transcriptional dynamics of leaf and the shoot apical meristem of soybean during different short-day treatment^28^. Our data showed that among the six soybean *FTs* identified, *GmFT7* displayed the highest expression in leaf following two short-day treatment (Fig. 4). To confirm the dynamics of *GmFT7* expression during the floral transition, we performed RT-PCR analysis using leaf and shoot apex samples collected at SD0 (0 short-day treatment) to SD4 (4 short-day treatment) at a 4 h interval. Four short-day treatment was chosen based on the appearance of *AP1* expression (a floral identity gene) in the SAM^32^. RT-PCR analysis showed that *GmFT7* mRNA had a consistent expression in leaf and shoot apex tissues in a 24 h cycle upon short-day treatment (Fig. 5). These results were consistent with the previous study, which suggested that *GmFT7* is ubiquitously expressed across different tissues^11^. In contrast, it has been shown that *GmFT2a* and *GmFT5a* mRNA expression follow circadian rhythm^12^.

### *Cis*-elements analysis

**Figure 6:**
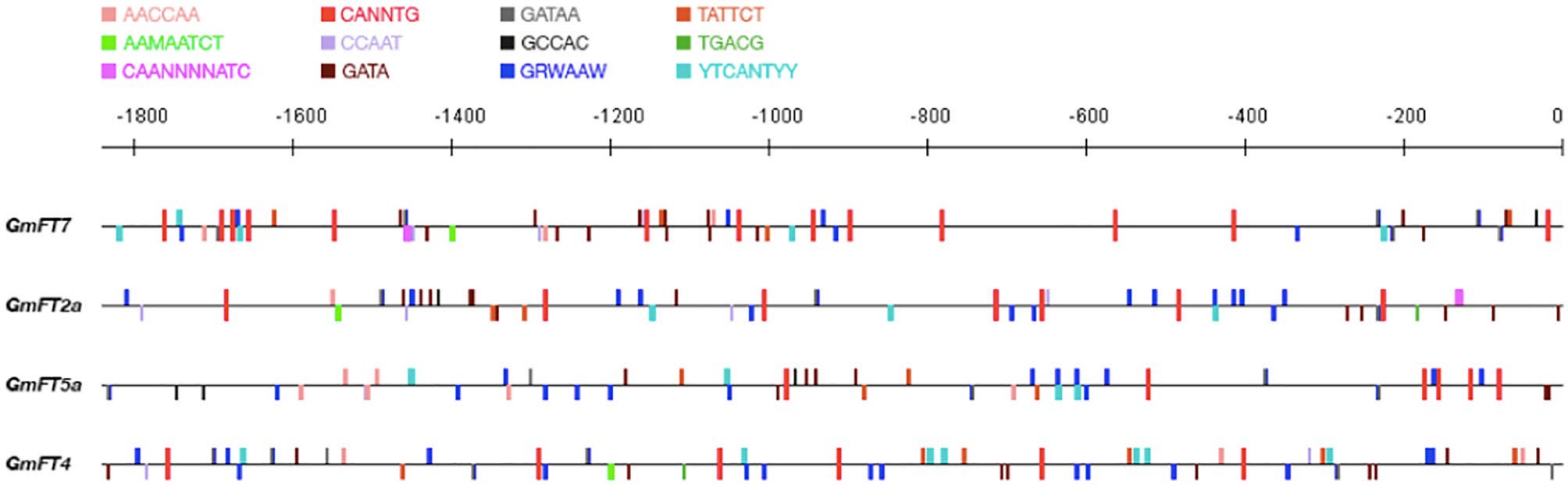
Light- and circadian clock-related cis-regulatory elements in *GmFT* promoters (1841 bp upstream of ATG). The cis-regulatory elements were identified by searching New PLACE database (https://www.dna.affrc.go.jp/PLACE/?action=newplace). Distribution of cis-regulatory elements was generated using TOUCAN (http://toucan.aertslab.org/software/toucan.php) software.

It is well established that *cis*-regulatory elements play essential roles in regulating gene expression. In eukaryotes, temporal and spatial gene expression is governed by the binding of transcription factors to *cis*-elements^33^. To compare the *cis*-elements between promoters of *GmFT7* and other flowering regulators such as *GmFT2a*, *GmFT5a* and repressor *GmFT4*, light- or circadian clock-related *cis*-elements were identified using NewPLACE webserver in 1.8 kb promoters upstream of ATG (Fig. 6). Unlike the *FT* promoter in *Arabidopsis* where a distal (approximately 5 kb upstream of ATG) promoter region contained critical elements for *FT* activity^34^, *GmFT7* had a promoter region of around 1.8 kb as determined by the presence of an upstream gene *Glyma.02G069400.1* (Supplementary Fig. S3). Twelve main *cis*-elements related to light or circadian-clock are summarised in Table 1. These include GATA, CIRCADIAN, GT1, I-Box, CCAAT, REalpha, E-Box, -10PE, INR, SORLIP1, CCA1, and ASF-1. Our analysis identified 11 out of 12 main *cis*-elements related to light or circadian-clock motifs in the promoter region of *GmFT7* (Table 1)*;* however, motif ASF-1 could not be located in the promoter region of *GmFT7.* It is worth noting that ASF-1motif only appeared once in *GmFT2a* and *GmFT4* promoter region. Among the 12 motifs, CCAAT^35,36^ and E-Box (CANNTG)^37^ had been suggested to have important roles in regulating the flowering process. CCAAT motifs are recognized by NUCLEAR FACTOR Y (NF-Y) proteins and its associated DNA complex^38^. NF-Y complex has been shown to participate in the flowering process in the photoperiodic regulation of flowering^39^. Research has shown that *Arabidopsis* cryptochrome 2-interacting basic helix-loop-helix 1 (CIB1) interacts with the chromatin region of *FT* promoter, which contains only E-Box elements other than G-Box (CACGTG) elements to mediate flowering control^37^.

**Table 1:** Summary of light- and circadian-clock related motifs in 1.8 kb soybean *GmFT* promoters.

Promoter	GATA	CIRCADIAN	GT1	I-Box	CCAAT	REalpha	E-Box	− 10PE	INR	SOELIP1	CCA1	ASF-1
*GmFT7*	20	1	13	6	3	4	26	4	5	1	1	0
*GmFT2a*	15	1	17	3	4	1	14	2	3	1	1	1
*GmFT5a*	12	0	22	5	5	6	12	4	4	3	0	0
*GmFT4*	17	0	24	7	2	3	12	6	7	0	1	1

The CCAAT motifs are the most frequent elements in the promoter region of eukaryotic genes typically found between 80 to 300 bp region upstream of the transcriptional start site. In this study, three CCAAT motifs were located in the proximal promoter region. Three CCAAT motifs in *GmFT7* promoter were located in a region around 1100–1500 bp upstream of the transcriptional start site. In contrast, four CCAAT motifs in *GmFT2a* promoter were scattered between 600 to 1800 bp upstream of ATG, while five and four CCAAT motifs were found in 1300–1650 bp region and 800–1800 bp region for *GmFT5a* and *AtFT* promoters, respectively. Interestingly, there were 26 E-Box motifs in *GmFT7* promoter, approximately two-fold than that of *GmFT2a/5a/4* promoters. E-box was found to be distributed throughout the promoter of *GmFT7*, while three CCAAT motifs exist in a 297 bp region (Fig. 6).

### Over-expression of *GmFT7* in *Arabidopsis*

**Figure 7:**
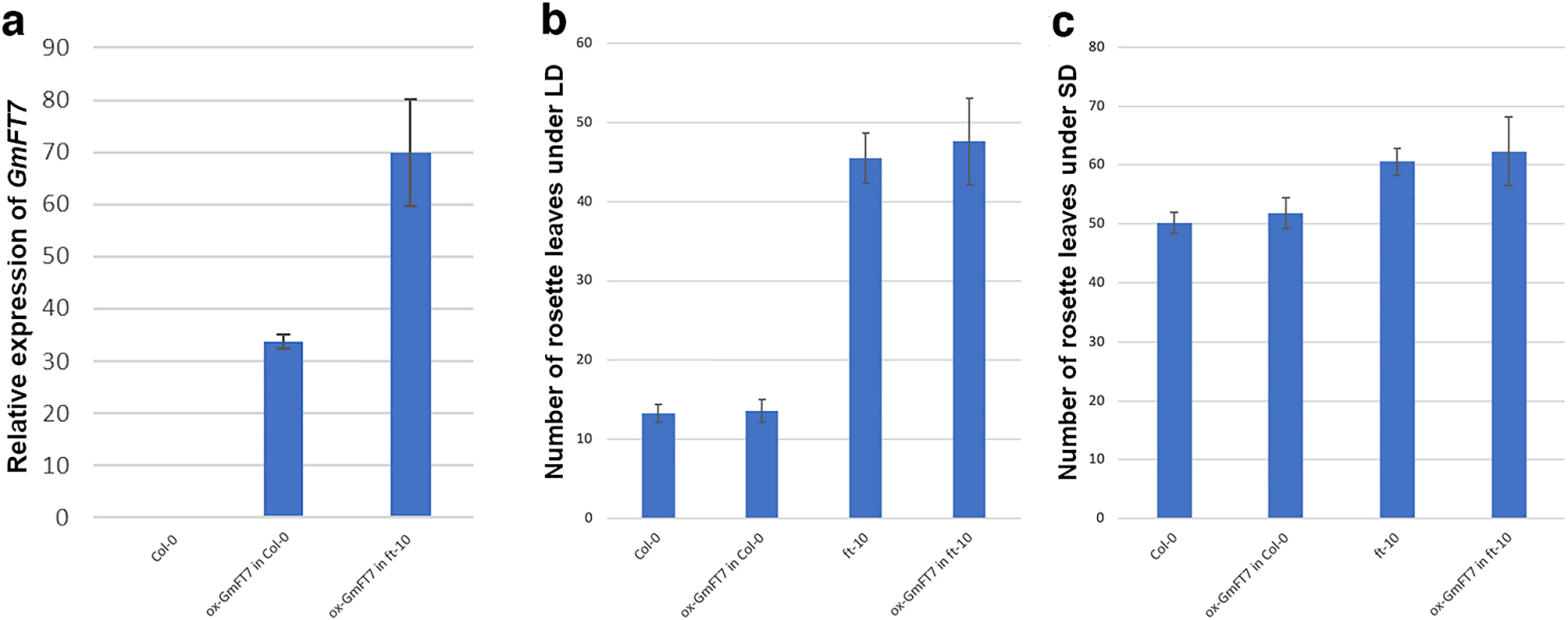
Analysis of transgenic *Arabidopsis* plants—flowering time and qPCR analysis. (**a**) Relative expression of ox-*GmFT7* in WT Arabidopsis and *ft-10* mutant background*.* Five independent plants for each of Col-0, ox-*GmFT7* in Col-0 and ox-*GmFT7* in *ft-10* were used for qPCR analysis along with two technical replicates. (**b**) Rosette leaves at bolting under LD conditions. (**c**) Rosette leaves at bolting under SD conditions. Error bars indicate standard deviation. Ten independent transgenic lines (5 plants from each independent line) were used for analysing transgenic plants, while 12 Arabidopsis plants were used as control (12 Col-0 plants and 12 *ft-10* mutant plants). *AT4G34270*^54^ was used as a reference gene. Relative gene expression was calculated using 2^−ΔΔCT^ method^55^. Results were shown as mean with standard deviation.

To investigate the role of *GmFT7* in flowering, we used an expression construct containing *GmFT7* CDS driven by a 35S promoter to transform WT Arabidopsis and complement or rescue the late-flowering mutant *ft-10* plant. The ectopic expression of *GmFT7* in the transgenic Arabidopsis was tested using qPCR. The results showed a successful expression of *GmFT7* in transgenic *Arabidopsis* plants (Fig. 7a). The transgenic *Arabidopsis* lines phenotypes were also evaluated under LD (16 h light/8 h dark) and SD (8 h light/16 h dark) conditions in this study. The statistical analysis of the rosette leaf number at the time of bolting showed that there were no significant differences (P > 0.05) between control and transgenic lines over-expressing *GmFT7* under inductive LD condition (rosette leaf number: 15.3 ± 1.4 in Col-0 versus 19.2 ± 2.2 in ox-*GmFT7* in Col-0; and 41.5 ± 3.2 in *ft-10* versus 46.8 ± 3.7 in ox-*GmFT7* in *ft-10* lines) or non-inductive SD condition (rosette leaf number: 50.2 ± 1.8 in Col-0 versus 51.8 ± 2.6 in ox-*GmFT7* in Col-0; and 60.6 ± 2.3 in *ft-10* versus 62.3 ± 5.8 in ox-*GmFT7* in *ft-10* lines) showing that *GmFT7* failed to influence flowering time in the transgenic plants (Fig. 7b,c). Further, transgenic lines harbouring *GmFT7* retained the late-flowering phenotype of Arabidopsis *ft-10* mutant background regardless of photoperiod (Fig. 7b,c), indicating that *GmFT7* could not complement the late-flowering *ft-10* mutants.

### Over-expression of *GmFT7* in soybean

**Figure 8:**
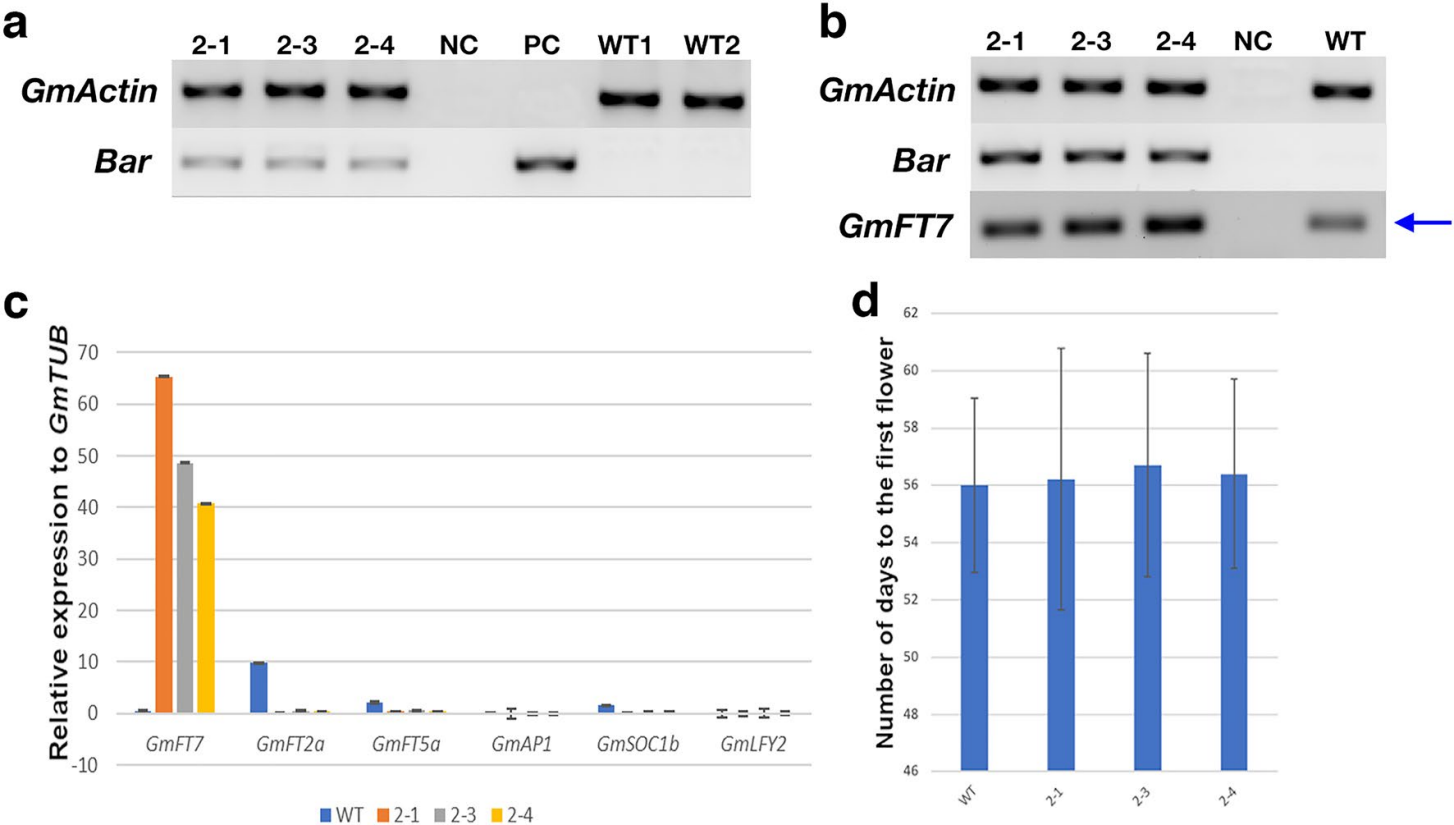
Analysis of transgenic soybean plants. (**a**,**b**) Transgenic soybean cv. Bragg plants over-expressing *GmFT7* analysis by genomic PCR showing the presence of the *Bar* gene (**a**) and RT-PCR confirmed the expression of both *bar* and *GmFT7* transcripts (**b**). Soybean reference gene *GmActin*, herbicide resistance *Bar* gene, and *GmFT7* CDS region were used for amplification. Trifoliate leaf from three T2 soybean transgenic lines; 2-1, 2-3 and 2-4 (21st day after sowing) was collected and used for genomic DNA and total RNA extractions. WT1, WT2 and WT, wild type soybean plants. NC, negative control (water was used as the template for PCR). PC, plasmid control (*35S:GmFT7:polyA* cassette in vector pUQC10255) as a positive control. (**c**,**d**) Flowering time and qPCR analysis. (**c**) qPCR analysis of *GmFT7* and other flowering related genes—*GmFT2a*, *GmFT5a*, *GmSOC1b*, *GmLFY2* and *GmAP1* in transgenic and wild-type soybean plants. Total RNA isolated from trifoliate leaves and shoot apex from plants (21st day after sowing) were used, Experiment had two biological replicates (pool of tissues from three plants were counted as one biological replicate) and two technical replicates. *GmTUB* was used as reference genes for soybean plants. Relative gene expression was calculated using 2^−ΔΔCT^ method^55^. Results were shown as mean with standard deviation from three replicates. (**d**) The number of days from sowing to first flower appearance in soybean. Three T2 lines (eight plants per line) were used for counting the number of days to first flower appearance.

Transgenic soybean plants over-expressing *GmFT7* were generated to investigate the function of *GmFT7* in the floral transition of soybean. The transgenic soybean plants were produced using *Agrobacterium*-mediated transformation described by Li et al.^40^ using glufosinate as a selectable agent. The transgenic status of the plants (T2 generation soybean) was confirmed by genomic PCR and expression of the *Bar*, and *GmFT7* genes were confirmed using RT-PCR (Fig. 8a,b). These experiments showed that the transgenic plants were harbouring and expressing the introduced genes (Fig. 8a,b). Further, *GmFT7* expression levels in these transgenic plants were determined using quantitative PCR. The qPCR results showed elevated expression of *GmFT7* in all the transgenic lines compared with WT plants (Fig. 8c). These plants were grown along with WT and flowering time was identified by recording the day of the first flower appearance. The flowering time for the transgenic plants and WT was recorded (Fig. 8d). The statistical analysis showed no significant difference (P > 0.05) in the flowering time between transgenic (56.2 ± 4.6, 56.7 ± 3.9 and 56.4 ± 3.3 in three transgenic lines) and wild type (56.0 ± 3.0) soybean plants (Fig. 8d).

It is well established that different flowering pathways are integrated by downstream genes such as LFY (LEAFY), SOC1 (SUPPRESSOR OF CONSTANS1), FT (FLOWERING LOCUS T). These integrators convey the outcomes to floral meristem identity gene, AP1 (APETALA 1) to evoke the floral initiation process at the shoot apical meristem. Accordingly, we studied the expression of *GmFT2a*, *GmFT5a*, *GmSOC1*, *GmLFY* and *GmAP1* in the transgenic and wild-type soybean plants via quantitative PCR. The expression patterns of these genes, as measured by qPCR, are shown in Fig. 8c. Higher expression of *GmFT7* was observed in the transgenic soybean lines. However, the expression of floral identity genes *GmAP1* and *GmLFY2* remained unaltered in the *GmFT7* over-expression lines (Fig. 8c).

## Discussion

Soybean is an important legume crop used for food and feed. Since soybean cultivars have a narrow latitude band that keeps it from adapting to the higher or lower latitudinal area, it would be desirable to breed soybean varieties adapted to a broader region. Such adapted lines would assist in increasing the soybean production to meet growing worldwide demand. Under the current changing climate scenario, it is especially crucial to ensure that soybean plants flower at the optimal time since flowering is a prerequisite for its seed production. Among the environmental factors that influence flowering, photoperiod is the most well-investigated. Several studies recently investigated soybean floral transition via bioinformatics and functional genomic approaches highlighting the roles of histone modifiers^41^ or circadian clock or photoperiod-related genes^42,43^. A comprehensive understanding of flowering signals such as FT, the florigen in the plant, could provide the crucial information on how legume plant like soybean coordinate both external and internal cues for the flowering process.

Soybean is a paleopolyploid crop plant with two genome duplications occurring about 59 and 13 million years ago, leading to multiple copies of three-fourths of its protein-coding genes^44^. Investigations on flowering time regulation in legumes, including soybean, have highlighted unique and conserved features^45^. As a result of these duplication events, the soybean genome contains at least ten *FT* homologues in soybean. Among them, *GmFT1a* and *GmFT4* were shown to be floral repressors^13,18^. On the other hand, *GmFT2a* and *GmFT5a* are flowering promoters^8,9,12^. *GmFT7* is a truncated portion (C-terminus) of *FT2c*. However, the evolution of *FT2c* is a soybean lineage-specific event which occurred after the separation of cultivated and wild soybeans^17^.

*GmFT7* shows high sequence similarity with *GmFT2a*, a soybean *FT* homologue which could promote flowering^12^. The *GmFT7* only harbours the fourth exon, and it retained the critical segment B and LYN in segment C^12,31^. Prediction of subcellular localization showed that *GmFT7* located in the same compartment as other FT homologues. Our analysis of the regulatory elements in its promoter region indicated similarities between other FTs—our data showed all the 12 *cis*-elements related to light or circadian-clock are present in the promoter region of *GmFT7*. Especially, CCAAT^35,36^ and E-Box that have been shown to play essential roles in flowering regulation. Though C-terminus of *GmFT7* resembles *GsFT2c* (Supplementary Fig. S1), *GsFT2c* could promote flowering in wild-type *Arabidopsis* and rescue the *ft-10* mutant^17^. In comparison, *GmFT7* could not promote flowering in wild-type *Arabidopsis* or compliment the late flowering *ft-10* mutants under inductive SD or non-inductive LD conditions. Transgenic soybean plants also showed that the overexpression of *GmFT7* could not promote flowering compared to wild type soybean plants. However, qPCR results showed that relative expression of *GmFT2a*, *GmFT5a* and *GmSOC1b* were decreased in transgenic soybean lines, but further work is required to show other possible players in the flowering pathways. To this end, CRISPR generated soybean plants would help unravel the floral network controlling the transition to flowering in soybean. However, our results indicate that *GmFT7,* which only carries the fourth exon, could not function as a floral signal in the soybean flowering process. We speculate that all the four exons in an *FT* homologue are required to promote the floral transition in soybean. It is also possible that due to soybean genome duplications and evolution *GmFT7* acquire the new role that needs further study.

## Methods

### Plant material and sampling

The soybean (cv. Bragg) plants were grown in a growth cabinet set at 16 h light/8 h dark (LD) or 8 h light/16 h dark (SD), 25 °C, 70% humidity and 400 μmol m^−2^ s^−1^ light intensity. The plants were grown from seeds for 10 days under LD and then transferred to SD. Leaf and shoot apex tissues were collected on a 4-h interval from LD10 (SD0) to SD4 for 5 days. For each time point, the samples were collected from 5 individual plants and pooled for RNA isolation. Samples were transferred in liquid nitrogen immediately following excision and stored at − 80 °C for further use.

Total RNA was isolated from leaf and shoot apex tissues using TRIzol reagent (Invitrogen, USA) as per the manufacturer’s instructions. The quality of RNA was examined by NanoDrop 1000 (Thermo Scientific, USA) and gel electrophoresis. According to the manufacturer, 1 μg of total RNA was used for first-strand cDNA synthesis using SuperScript III Reverse Transcriptase (Thermo Fisher, USA) in a 20 μL reaction’s protocol. After reverse transcription, 80 μL of nuclease-free water was added to dilute the cDNA. 2 μL was then used for PCR reaction. *GmActin* was used as a reference gene.

### Bioinformatics analysis

Soybean FT homologues and *Arabidopsis* FT protein sequences were retrieved from Phytozome (https://phytozome.jgi.doe.gov/pz/portal.html; version 12.0) database. Sequence alignment was performed using MUSCLE^46^ program in MEGA7^47^ with default parameters. The phylogenetic tree was inferred using the Likelihood method with cpREV + G substitution model; partial deletion was used for Gaps/Missing data treatment. Reliability was examined using the Bootstrap method with 1000 replicates. A phylogenetic tree was drawn using FigTree (http://tree.bio.ed.ac.uk/software/figtree/; version 1.4.3). Theoretical pI, molecular weight, instability, and GRAVY values were calculated using the ProtParam tool (https://web.expasy.org/protparam/). Protein subcellular localization was analyzed using a predictive tool, ProtComp 9.0 (http://www.softberry.com).

Gene structures of soybean and *Arabidopsis FT* genes were displayed by Gene Structure Display Server (GSDS) 2.0^48^. *Cis*-regulatory elements were examined using NewPLACE web server^49^. Location of *cis*-elements was highlighted using TOUCAN 2^50^. Protein sequences of GmFT1a/2a/4/5a/7 and AtFT were aligned using Clustal Omega^51^. The alignment was imported to GeneDoc^52^ (version 2.7.000) for shading.

### Plant constructs and transformation

For gene expression construct, the coding sequence of *Glyma02g07650* (*GmFT7*) was amplified (with XhoI and BamHI restriction enzyme sites on 5′ and 3′ end, respectively) and ligated to pGEM-T Easy vector (Promega). The vector was digested, and gene fragment was ligated to pRT101 at Xho I and Bam HI sites, to obtain 35S promoter and polyA terminator. Then the *35S:GmFT7:polyA* cassette was excised using Hind III and ligated to pUQC10255 expression vector (Supplementary Fig. S4). Transformation of *Arabidopsis* Col (WT) and *ft-10 mutant* was performed using the floral dip method^53^.

Bragg cultivar was used for soybean transformation following the protocol by Li et al.^40^ with modifications; selection on SIM medium containing 4 mg/L glufosinate (Sigma, USA) was started during the second subculture step SIM and continued till SEM medium. 100 mg/L Cefotaxime (GoldBio, USA), 50 mg/L Vancomycin (GoldBio, USA) and 50 mg/L Ticarcillin (GoldBio, USA) were used instead of carbenicillin on SIM, SEM and RM medium. Five independent experiments were conducted using an average of 110 seed explants per experiment and recovered five transgenic shoots. However, one-line produced seeds. The transgenic seeds were multiplied and selected for the *Bar* gene. Three T2 lines were used for analysis.

### Confirmation of transgenic soybean plants

Trifoliate leaf (21st day after sowing) was collected and used for genomic DNA and total RNA extractions. Genomic PCR and RT-PCR were performed to detect *Bar* gene and *GmFT7* expression in the putative transgenic soybean plants. Genomic DNA was extracted using the CTAB method (https://agriculture.uq.edu.au/files/5650/plant-genomic-dna-extraction-by-ctab-2-fiona.pdf). 100 ng of genomic DNA was used for PCR reaction. Genomic PCR and RT-PCR were performed at 94 °C for 3 min, followed by 35 cycles of 94 °C for 45 s, 50 °C for 30 s and 72 °C for 60 s, with a final extension for 10 min at 72 °C. Supplementary Table S1 lists all the primers used in this study.

### Flowering time determination

Flowering time in *Arabidopsis* was determined by counting rosette leaves both under LD and SD conditions. Ten independent Arabidopsis transgenic lines (5 plants from each independent line) were used for analysing transgenic plants, while 12 Arabidopsis plants were used as control (12 Col-0 plants and 12 *ft-10* mutant plants). Soybean plants were grown under LD for 4 weeks before shifting to SD conditions. Flowering time in soybean was identified by recording the day of the first flower appearance. Three T2 lines (8 plants per line) were used for counting the number of days to first flower appearance. Significant differences were tested using Student’s *t*-test in Microsoft Excel.

### Quantitative PCR (qPCR)

Five independent plants for each of Col-0, ox-*GmFT7* in Col-0 and ox-*GmFT7* in *ft-10* were used for qPCR analysis with two technical replicates. For soybean qPCR analysis, two biological replicates of trifoliate leaf and shoot apex tissues (pool of tissues from three plants were counted as one biological replicate) and two technical replicates were used. Total RNA isolated from a trifoliate leaf from plants (21st day after sown) were used for the relative expression of *GmFT7*, *GmFT2a* and *GmFT5a*, while total RNA extracted from shoot apex were used for examining the expression of *GmAP1*, *GmSOC1b* and *GmLFY2*. qPCR was performed using Brilliant III Ultra-Fast SYBR Green QPCR Master Mix (Agilent Technologies, USA) on a Stratagene Mx3005P qPCR platform (Agilent Technologies, USA). 2 µL cDNA was used as a template in a 10 µL qPCR reaction (5 µL 2xSYBR Green Master Mix, 0.3 µL of each primer (final concentration of 300 nM), 0.3 µL diluted (1:500) reference dye and 2.1 µL nuclease-free water). The qPCR program was 95 °C for 3 min, followed by 40 cycles of 20 s at 95 °C and 20 s at 60 °C, then finished with 1 min at 95 °C, 30 s at 55 °C and 30 s at 95 °C for melting curve. Primers (Supplementary Table S1) were retrieved from previous studies (*GmTUB*, *GmFT2a*, *GmFT5a*, *GmAP1*, *GmSOC1b*, and *GmLFY2*)^14^. *GmTUB* and *AT4G34270*^54^ were used as reference genes for soybean and Arabidopsis plants, respectively. Relative gene expression was calculated using 2^−ΔΔCT^ method^55^.

## Supplementary Information

Supplementary Information.
