# Supplementary material for: Molecular characterization of a soybean FT homologue, GmFT7
Source: Sci Rep. 2021 Feb 11;11:3651. doi: 10.1038/s41598-021-83305-x (PMC7878754; doi:10.1038/s41598-021-83305-x)
Supplement: Supplementary file 1 — Supplementary Information. [file 41598_2021_83305_MOESM1_ESM.pdf]

Supplementary Information

Molecular characterization of a soybean *FT* homologue, *GmFT7*

Senhao Zhang[1], Mohan B. Singh[1] and Prem L. Bhalla[1*]

1. Plant Molecular Biology and Biotechnology Laboratory, School of Agriculture and Food, Faculty of Veterinary and Agricultural Sciences, The University of Melbourne, Parkville, Victoria 3010, Australia.

[*]Corresponding author: Prem L. Bhalla (premlb@unimelb.edu.au)

```
GmFT7            1 --------------------------------------------------      0

GsFT2c           1 MPSGSRDPLVVGRVIGDVLDPFECSIPMRVSYNNRDVSNGCEFKPSQVVN     50

GmFT7            1 --------------------------------------------------      0

GsFT2c          51 QPRINIGGDDLRNFYTLIAVDPDAPSPSDPNLREYLHWLVTDIPATTGPS    100

GmFT7            1 -------------MMGIHRLVFVLFRQLGRETVYAPGWRQNFNTREFAEL     37
                                |||||||||||||||||||||||||||||||||||||
GsFT2c         101 FGHEVVTYESPRPMMGIHRLVFVLFRQLGRETVYAPGWRQNFNTREFAEL    150

GmFT7           38 YNLGLPVAAVYFNIQRESGSGGRRLYH     64
                   |||||||||||||||||||||||||||
GsFT2c         151 YNLGLPVAAVYFNIQRESGSGGRRLYH    177
```

Supplementary Figure 1. Pairwise protein sequence alignment of GmFT7 and GsFT2c.

Protein sequences of soybean (*Glycine max*) GmFT7 (Glyma02g07650) and wild soybean (*Glycine soja*) GsFT2c (XP_028198760) were retrieved from Phytozome (v12; https://phytozome.jgi.doe.gov/pz/portal.html#) and NCBI, respectively. Pairwise protein sequence alignment was performed using Needle (https://www.ebi.ac.uk/Tools/psa/emboss_needle/) with default parameters.

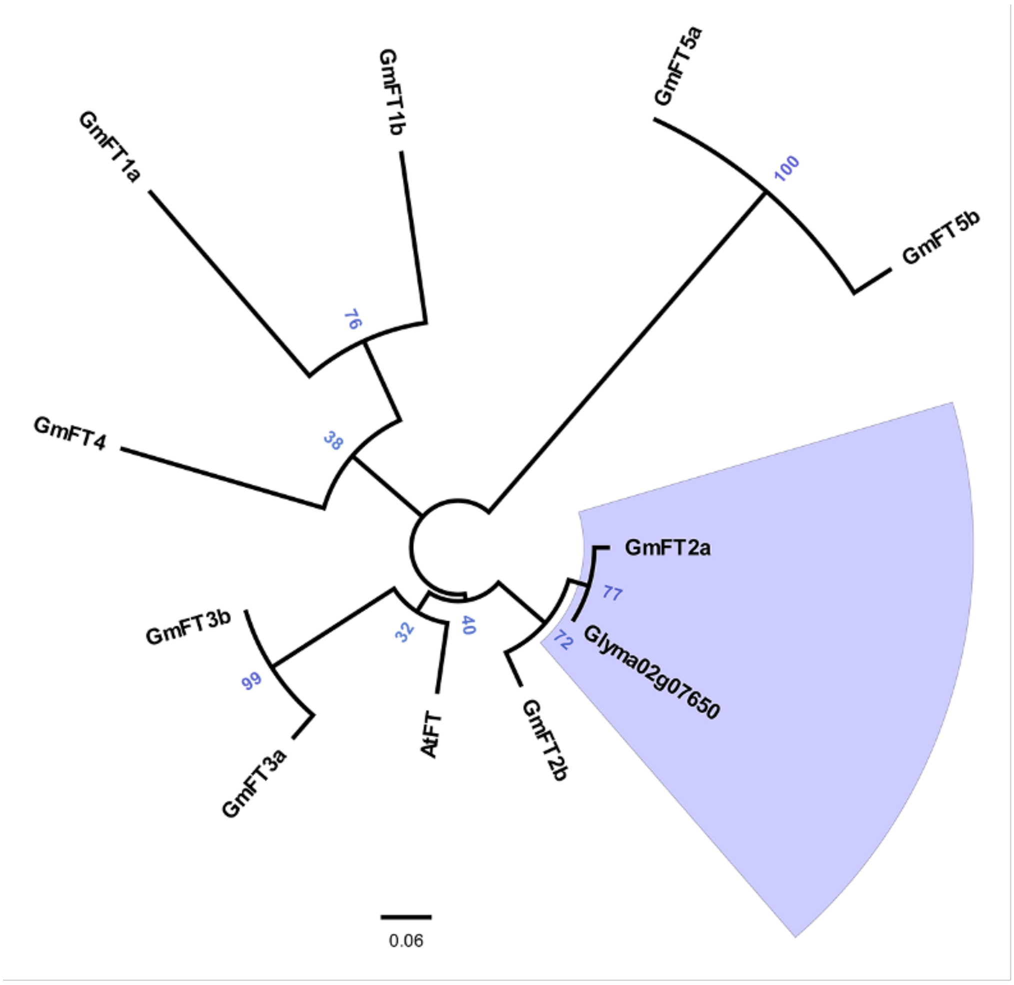

Supplementary Figure 2. Phylogenetic tree of FT homologues.

Protein sequences of ten GmFT homologues and Arabidopsis FT were retrieved from Phytozome (v12; https://phytozome.jgi.doe.gov/pz/portal.html#). Maximum Likelihood tree was constructed using MEGA7.

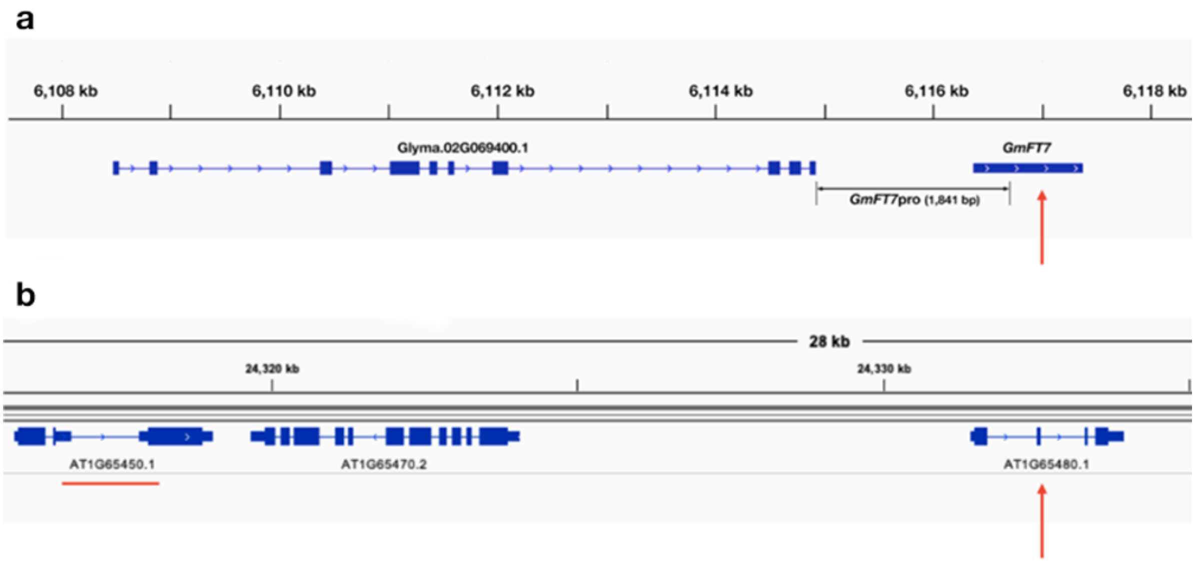

Supplementary Figure 3. Locations showing distances with the upstream genes of soybean *GmFT7* (a) and Arabidopsis *FT* (b) on their corresponding chromosomes.

1.8 kb promoter for *GmFT7* was chosen because there is another gene (*Glyma.02G069400.1*) located upstream of *GmFT7* (a). From the end of *Glyma.02G069400.1* to the ATG of *GmFT7*, the length is 1841 bp. Hence, the promoter of *GmFT7* was cloned as 1841 bp. On the other hand, Arabidopsis, *AtFT* (*AT1G65480*, + strand) is located downstream of gene *AT1G65450* (+strand). The distance between *AtFT* and its closest upstream gene *AT1G65450* is more than 10 kb (b). Locations on chromosomes were displayed using Integrative Genomics Viewer (IGV) (http://software.broadinstitute.org/software/igv/home).

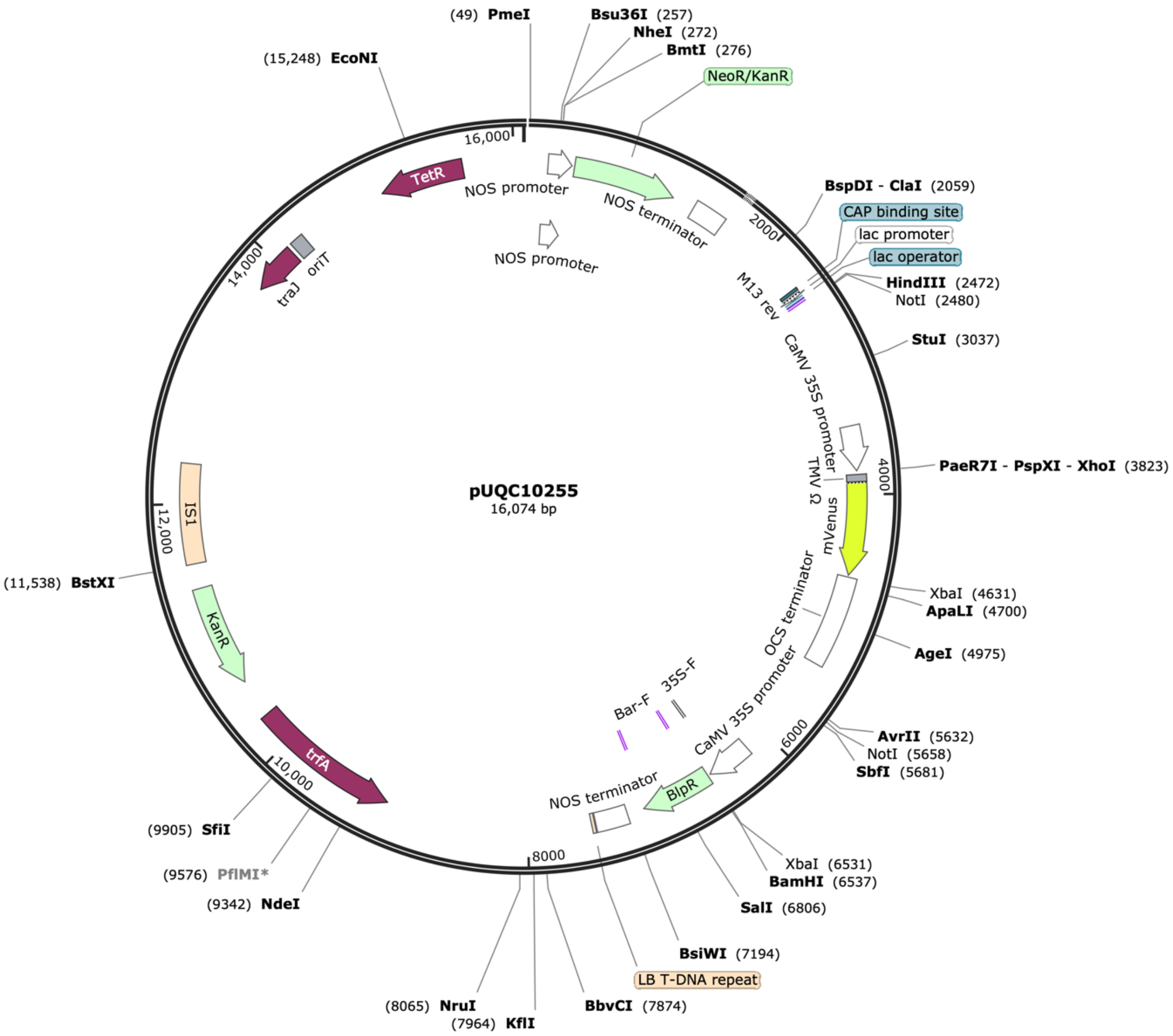

Supplementary Figure 4. Map of the expression vector pUQC10255 used in this study.

The map of the expression vector pUQC10255 is displayed using SnapGene Viewer 5.2.3 (https://www.snapgene.com/snapgene-viewer/). Main features of pUQC10255 are labelled accordingly.

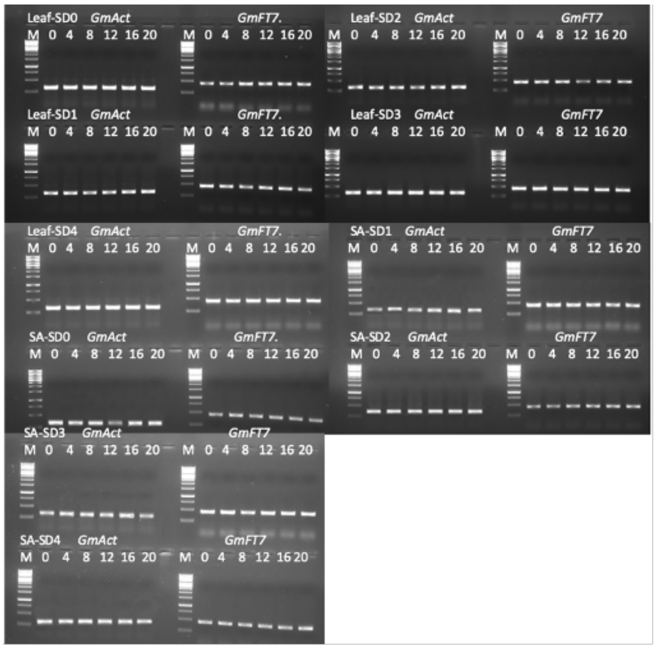

Supplementary Figure 5. Original gel electrophoresis pictures for Fig. 5.

M, GeneRuler 1kb DNA Ladder (Thermo Fisher, Cat. Number: SM0311). SA, shoot apex. SD, short day. *GmAct*, soybean reference gene *Actin*.

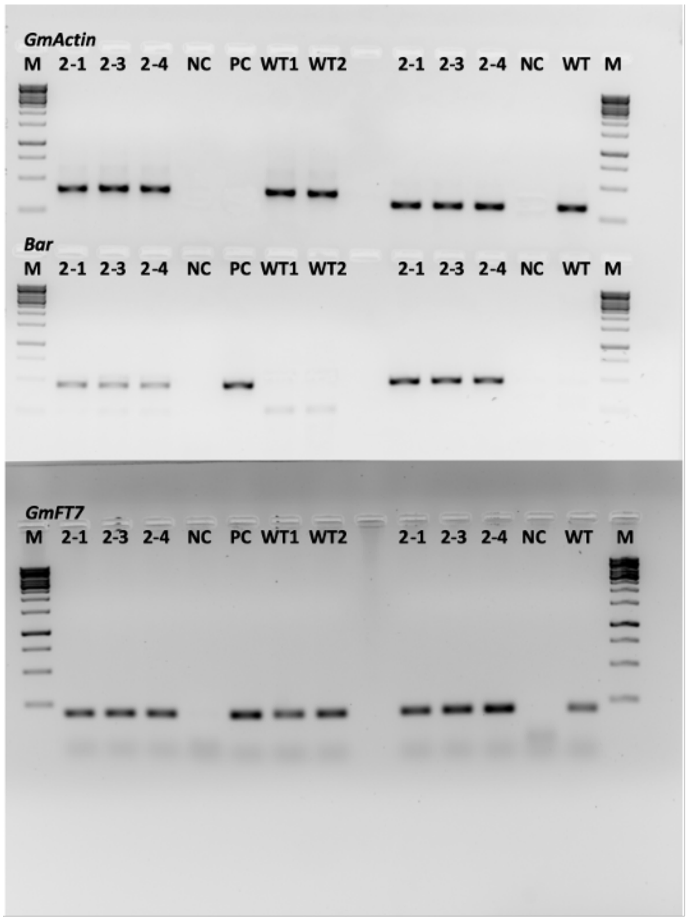

Supplementary Figure 6. Genomic PCR (a) and RT-PCR (b) to confirm transgenic status of soybean plants.

This is the original gel picture for Fig. 8a/b in the main manuscript. Transgenic soybean plants over-expressing *GmFT7* in soybean cv. Bragg were used.Genomic PCR (upper panel) confirmed the presence of *Bar* gene while RT-PCR (lower panel) analysis showed the expression of the *Bar* and *GmFT7* transcripts. Soybean reference gene *GmActin*, herbicide resistance *Bar* gene, and *GmFT7* CDS region. 2-1, 2-3 and 2-4, three individual T2 transgenic soybean plants. WT1, WT2 and WT, wild type soybean plants. NC, negative control (water was used as template for PCR). PC, plasmid control (*35S:GmFT7:polyA* cassette in vector pUQC10255) as a positive control. M, GeneRuler 1kb DNA Ladder (Thermo Fisher, Cat. Number: SM0311).

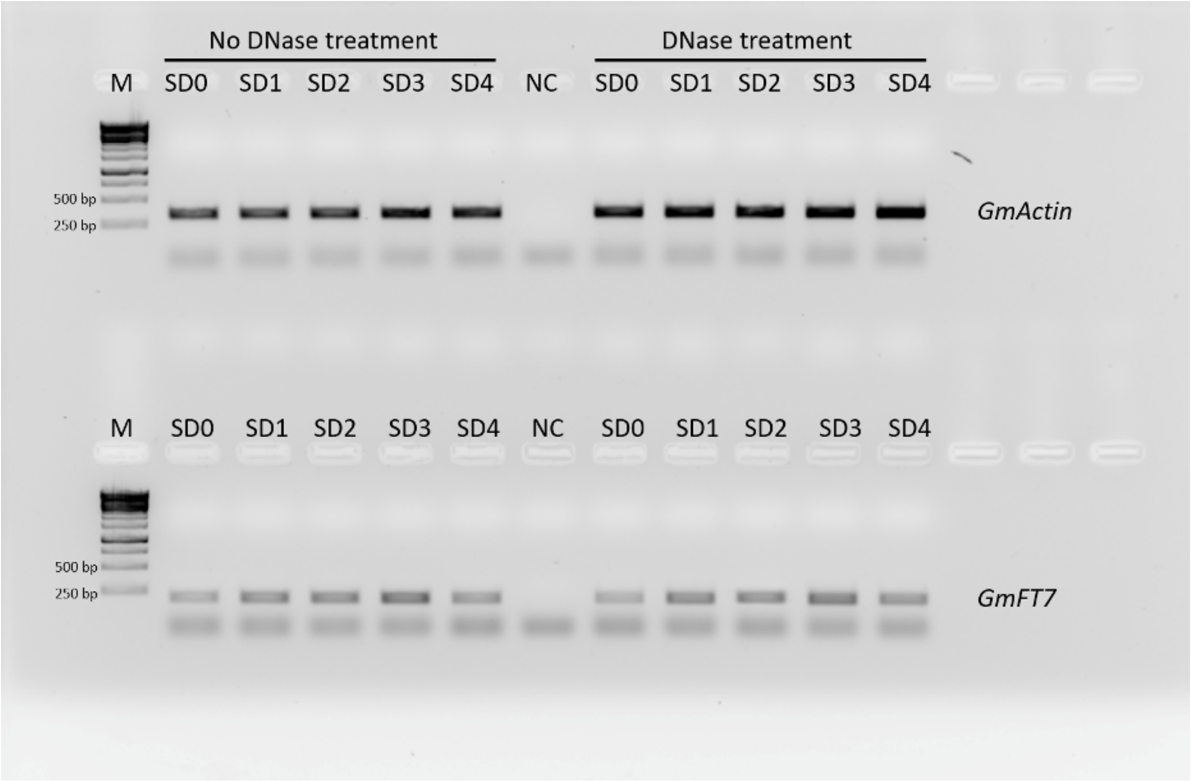

Supplementary Figure 7. Expression of *GmFT7* using PCR.

Total RNA was extracted using TRIzol and followed by "without DNase treatment" to compare the PCR results with previous PCR experiments we performed. M, GeneRuler 1kb DNA ladder (Thermo Fisher); SD0-SD4, 0 to 4-SD treatment. *GmActin*, reference gene for PCR. NC, negative control. DNase treatment, total RNA was treated using DNase to remove potential genomic DNA contamination (previous RT-PCR on *GmFT7* we performed); No DNase treatment, total RNA was not treated with DNase for potential genomic DNA removal.

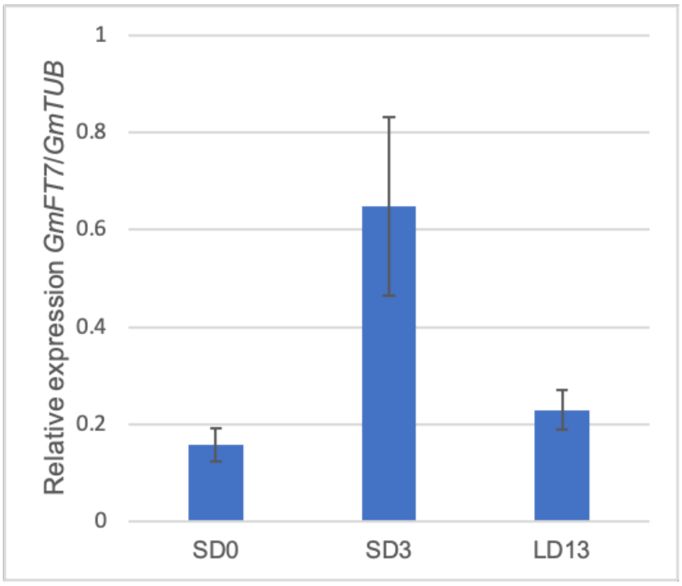

Supplementary Figure 8. Relative expression of *GmFT7* under SD0, SD3 and LD13.

qPCR was performed to check *GmFT7* expression on SD3 and LD13. Bar chart was shown as mean and standard deviation with three replicates. *GmTUB* was used as a reference gene for normalization. Student's T-test was performed in R (https://www.r-project.org/) and it showed that the expression of *GmFT7* was higher under SD3 compared to LD13, however the difference was not significant (p-value = 0.2142). While expression of *GmFT7* was significantly ($p < 0.05$) higher under SD3 compared to SD0.

Supplementary Table 1. Primers used in this study.

**Name**	**Sequence (5' -> 3')**	**Length (nt)**	**Note**
GmFT7mRNA-F	CCGTCGTTAAAAGCACTTAATAACCG	26	*GmFT7* full-length mRNA
GmFT7mRNA-R	CCCCATAATTTAACTTGTGCTCAAG	25	
FT7-cloF	AATGCTCGAGATGATGGGGATTCATCGTTTA	31	Cloning of *GmFT7* CDS
FT7-cloR	CGCGGATCCTCAATGGTATAACCTTCTTC	29	
GmFT7-1F	ATGATGGGGATTCATCGTTTAGTG	24	Amplification of *GmFT7* CDS;
GmFT7-1R	TCAATGGTATAACCTTCTTCCACC	24	qPCR fpr *GmFT7*
GmActin-F	ATCATGTTTGAGACCTTCAATGTG	24	Reference gene *GmActin*
GmActin-F	CTCGAGTTCTTGCTCATAATCTAGG	25	
BAR-1F	GTACCGGCAGGCTGAAGTCC	20	*Bar* gene
BAR-1R	CGGTCTGCACCATCGTCAAC	20	
AT4G34270-F	GTGAAAACTGTTGGAGAGAAGCAA	24	qPCR reference gene for
AT4G34270-R	TCAACTGGATACCCTTTCGCA	21	Arabidopsis
GmTUB-q-F	GAGAAGAGTATCCGGATAGG	20	qPCR reference gene for
GmTUB-q-R	GAGCTTGAGTGTTCGGAAAC	20	Soybean
GmFT2a-q-F	GGATTGCCAGTTGCTGCTGT	20	qPCR
GmFT2a-q-R	GAGTGTGGGAGATTGCCAAT	20	
GmFT5a-q-F	GCCTTACTCCAGCTTATACT	20	qPCR
GmFT5a-q-R	GGCATGCTCTAGCATTGCAA	20	
GmAP1-q-F	TGAACATGGGTGGCAATTAC	20	qPCR
GmAP1-q-R	TGTCAAATGCCATACCAAAG	20	
GmSOC1b-q-F	AAGAAGCCCAACTGCAATGT	20	qPCR
GmSOC1b-q-R	GGGCTTCAGAAATGAGGAAAGG	22	
GmLFY2-q-F	TGACGAAGGAAACATTAACACTGG	24	qPCR
GmLFY2-q-R	GCCTGAACCTGCATCAAGAA	20	

Note:

Primers for *GmFT7* mRNA in Fig. 5 were GmFT7-1F/GmFT7mRNA-R.

Primers for *GmFT7* in genomic PCR and RT-PCR (Fig. 8) were GmFT7-1F/ GmFT7-1R to amplify *GmFT7* CDS.
